# Surgical resection of metachronous hepatic metastases from gastric cancer improves long-term survival: A population-based study

**DOI:** 10.1371/journal.pone.0182255

**Published:** 2017-07-31

**Authors:** Szu-Chin Li, Cheng-Hung Lee, Chung-Lin Hung, Jin-Chia Wu, Jian-Han Chen

**Affiliations:** 1 Department of Oncology, Dalin Tzu Chi Hospital, Buddhist Tzu Chi Medical Foundation, Chiayi, Taiwan; 2 School of Medicine, Tzu Chi University, Hualien, Taiwan; 3 Department of General Surgery, Dalin Tzu Chi Hospital, Buddhist Tzu Chi Medical Foundation, Chiayi, Taiwan; 4 Department of General Surgery, E-Da Hospital, Kaohsiung, Taiwan; 5 School of Medicine, I-Shou University, Kaohsiung, Taiwan; National Cancer Center, JAPAN

## Abstract

**Introduction:**

Hepatic metastases are diagnosed synchronously in 3–14% of patients with gastric cancer, and metachronously in up to 37% of patients following ‘‘curative” gastrectomy. Most patients who have gastric cancer and hepatic metastasis are traditionally treated with palliative chemotherapy. The impact of liver resection is still controversial. We attempted to assess whether liver resection can improve survival in cases of metachronous hepatic metastases from gastric cancer through a nationwide database.

**Materials and methods:**

We conducted a nationwide cohort study using a claims dataset from Taiwan’s National Health Insurance Research Database (NHIRD). We identified all patients with gastric cancer (diagnostic code ICD-9: 151.x) from the Registry for Catastrophic Illness Patient Database (RCIPD) of the NHIRD who received gastrectomy and as well as those with metachronous (≥180 days after gastrectomy) liver metastases (ICD-9 code: 197.7) between 1996/01/01 and 2012/12/31. Patients with other malignancies, with metastasis in the initial admission for gastrectomy and with other metastases were excluded. They were divided into two groups, liver resection group and non-resection group. All patients were followed till 2013/12/31 or withdrawn from the database because of death.

**Results:**

653 patients who fullfilled the inclusion criteria were included in the research. They were divided into liver resection group (34 patients) and non-resection group (619 patients). There were no differences between the two groups in gender, Charlson Comorbidity index and major coexisting disease. Kaplan-Meier analysis demostrated the liver resection group had significantly better overall survival than the non-resection group. (1YOS: 73.5% vs. 19.7%, 3YOS: 36.9% vs. 6.6%, 5YOS: 24.5.3% vs. 4.4%, *p* <0.001). After COX analysis, the liver resection group showed statistical significance for improved patient survival (HR = 0.377, 95%CI: 0.255–0.556. p<0.001).

**Conclusion:**

Liver resection in patients presenting with metachronous hepatic metastases as the sole metastases after curative resection of gastric cancer is associated with a significant survival improvement and should be considered a treatment option for such patients.

## Introduction

Gastric carcinoma is the fourth most common type of tumor globally[1] and the second leading cause of cancer-related death worldwide[2]. It is more common in the Far East than in the West[3]. At the time of diagnosis, 35% of patients have distant metastases and 4–14% have metastasis to the liver[4]. Hepatic metastases are diagnosed synchronously in 3–14% of patients with gastric cancer[5], and metachronously in up to 37% of patients following ‘‘curative” gastrectomy[6]. Approximately 80% of metachronous metastases after curative gastrectomy appear within the first two postoperative years[7].

In fact the survival benefit of hepatic resection for either synchronous or metachronous gastric hepatic metastases remains debatable[8]. Neoadjuvant chemotherapy, by shrinking locally advanced cancers prior to surgical resection, enhances the opportunity for local treatment and improves long-term survival[9]. Patients who have gastric cancer and hepatic metastasis are traditionally treated with palliative chemotherapy, which has been shown to be superior to supportive care by improving median survival from 4.3 to 12 months[10]. However, surgery is not always an option if there are hepatic metastasis[6], only 0.4–1% of metastatic gastric cancer patients are eligible for radical surgery[11]. Moreover, therapeutic plans to deal with these metastases can vary between surgeons, especially in the absence of institutional guidelines or protocols.

In this nationwide cohort study, we used claims data from Taiwan’s National Health Insurance Research Database (NHIRD) to evaluate the impact of liver resection on the survival of patients with metachronous hepatic metastases from gastric cancer. The secondary aim was to assess change in overall survival since 2009, when Taiwan’s National Health Insurance began to cover the chemotherapy regimen XELOX (Capecitabine and Oxaliplatin) for gastric cancer with metachronous hepatic metastases. We attempted to assess whether liver resection can improve survival in cases of metachronous hepatic metastases from gastric cancer.

## Materials and methods

### Database and study sample

We conducted a nationwide cohort study using a claims dataset from Taiwan’s National Health Insurance Research Database (NHIRD) (registered number NHIRD-103-246). The data are provided by the National Health Insurance Administration and the Ministry of Health and Welfare. Data in the NHIRD that could be used to identify patients is scrambled before being sent to the National Health Research Institutes for database construction and is further scrambled before being released to each researcher. The protocol of this study was fully reviewed and approved by the Institutional Review Board of Buddhist Dalin Tzu Chi Hospital (B10503009).

In this database, ICD-9 codes are assigned to the diagnosis made and procedures used during each admission. We extracted the data on registry for catastrophic illness patient Database (RCIPD) and inpatient expenditures by admission (DD) from the NHIRD database from January 1, 1996, to December 31, 2013 for our study.

### The inclusion criteria

We identified all patients with gastric cancer (diagnostic code ICD-9: 151.x) from the Registry for Catastrophic Illness Patient Database (RCIPD) of the NHIRD [12]. Surgical pathological confirmation of gastric cancer was required for patients to be registered in the RCIPD. Admission data regarding inpatient expenditures by admissions (DD) from the NHIRD database were extracted. All patients admitted between 1996/01/01 and 2012/12/31 and discharged with gastric cancer (ICD codes 151.x) who received subtotal or total gastrectomy (ICD-9 procedure codes: 40.29, 40.3, 40.52, and 40.59) were extracted. Patients who developed liver metastasis during followed-up period were included. Patients with liver metastasis were identified by diagnosis code (ICD-9: 197.7). The date of first admission for liver metastasis after gastrectomy was defined as the date of liver metastasis detection. Metachronous liver metastasis was defined as liver metastasis identified at least 180 days after the index admission date[13].

### The exclusion criteria

Patients who developed liver metastasis within 180 days after admission for gastrectomy, who developed other malignancies or other metastases before or during the admission for liver metastasis, or who developed liver metastasis after 2012/12/31 were excluded.

### Study cohort

**Fig 1:**
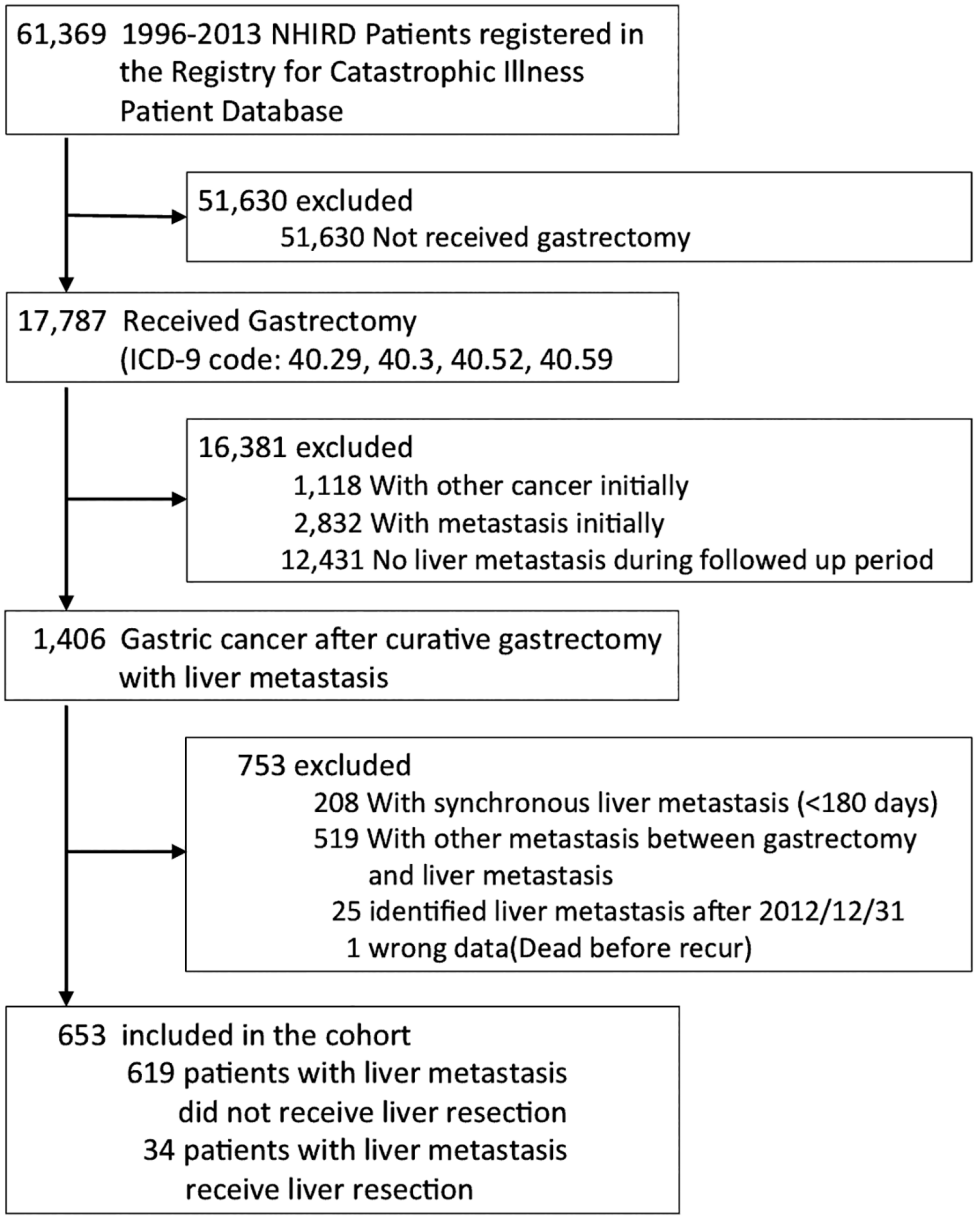
The criteria for selecting study patients.

All gastrectomized patients with newly diagnosed gastric cancer without initial metastasis, and identified metachronous liver metastasis between 1996/01/01 and 2012/12/31 were divided into two groups based on whether or not they received liver resection (ICD-9 procedure code: 50.29, 50.3). The patient selection algorithm is shown in Fig 1.

### Primary end point

The primary end point was death from any cause. Death data were obtained from the RCIPD. If the date of death was not available in the RCIPD, then death was defined as withdrawal from the NIH program[12]. All patients were followed until their death or the end of the study period (i.e., December 31, 2013).

### Secondary end point

Capecitabine and Oxaliplatin (XELOX) was covered as a salvage chemotherapy regimen by the national health insurance since 2009. As such we were able to evaluate the effect of the new regimen by separating our patients into 4 groups, including two liver resection groups (one after and one before 2009) and two non-resection groups (one after and one before 2009).

### Covariant assessment

Comorbidities were identified by ICD code in the NHIRD database records. The Charlson comorbidity index (CCI) [14, 15] except malignancies and metastasis of all patients were used for the health status assessment. Comorbidities included diabetes mellitus (250), hypertension (401–405), liver disease (571.2, 571.4–6, 572.2–8, 456.0–456.21), renal disease (582, 583, 585, 586, 588), peptic ulcer disease (531–534), chronic pulmonary disease (490–496, 500–505, 506.4), cerebrovascular disease (430–438), and myocardial infarction (410, 412) were identified as covariates.

### Statistical analysis

We used SPSS software (IBM, Chicago, IL, USA) for descriptive statistics and contingency tables for data analysis. The chi-square test was used to compare the categorical variables such as age groups, gender, and comorbidities between 2 groups and generated the contingency table. The Student *t* test and Mann Whitney *U* test were used for continuous variables. The Kaplan–Meier method with log-rank test was used to compare differences in overall survival after identification of liver metastasis between surgical and non-surgical groups. All variables with p value less than 0.2 were entered into a backward stepwise Cox proportional hazards model to calculate the hazard ratio. A p-value of <0.05 was considered statistically significant.

## Results

We identified 61,369 patients with gastric cancer from the RCIPD, and only 17,787 of these patients received gastrectomy. Of the 13,837 patients remaining after excluding 1,118 patients who developed other cancers and 2,832 patients who developed metastasis before or during the admission for gastrectomy, only 1,406 patients developed liver metastasis. In all, 753 of these patients were excluded (208 patients with synchronous liver metastasis [< 180 days after admission for gastrectomy], 519 patients who developed other metastasis between admissions for gastrectomy and liver metastasis, 25 patients with liver metastasis identified after 2012/12/31, and 1 patient with incorrect data, who died prior to the last admission date). As a result a total of 653 patients with gastrectomy and metachronous liver metastasis were included in this study.

These patients were separated into a non-resection group and a liver resection group. Table 1 compares the clinical characteristics, cormorbidities, and follow-up duration between the two groups. Compared to patients in the resection group, patients in the non-resection group were older (68.62 ± 12.7 vs 62.03 ± 14.4 years, p = 0.007) but similar in gender distribution; had significantly shorter total follow-up duration (31.68 ± 29.26 vs 58.08 ± 34.54 months: p<0.001); similar mean time from gastrectomy to detection of liver metastasis (22.98±21.00 vs 23.94±15.59 months, p = 0.105); significantly shorter mean follow-up time from identification of liver metastasis to the end point (9.75±21.39 vs 33.58±31.39 months, p<0.001), and similar comorbidities and CCI score.

**Table 1:** The basic characteristics of the two groups.

Clinical characteristics	Non-resection group	Liver resection Group	*p*
	(N = 619)	(N = 34)	
**Age, mean (SD) y**	68.62 (12.7)	62.03 (14.4)	0.007
**Gender**			0.428
Male	457 (73.8%)	23 (67.6%)	
Female	162 (26.2%)	11 (32.4%)	
**Total follow up time (Month)**			<0.001
Mean (SD)	31.68 (29.96)	58.08 (34.54)	
Median (range)	22 (6–206)	49 (7–107)	
**Time to liver metastasis detection (Month)**			0.105
Mean (SD)	22.98 (21.00)	23.94 (15.59)	
Median (range)	15 (5–163)	20 (6–73)	
**Operation timing**			
Immediate		31 (91.2%)	
Delayed		3 (8.8%)	
**Follow up duration after liver metastasis detection (Month)**			<0.001
Mean (SD)	9.75 (21.39)	33.58 (31.39)	
Median (range)	3.13 (0.03–196.46)	24.80 (0.333–118.10)	
**Major coexisting disease**			
DM	77 (12.4%)	4 (11.8%)	1.000
HTN	103 (16.6%)	8 (23.5%)	0.345
Liver disease	66 (10.7%)	2 (5.9%)	0.239
Chronic pulmonary disease	37 (6.0%)	1 (2.9%)	1.000
Cerebrovascular disease	19 (3.1%)	1 (2.9%)	1.000
Myocardial infarction	4 (0.6%)	0	1.000
Peptic ulcer disease	124 (20.0%)	6 (17.6%)	1.000
Renal disease	9 (1.5%)	1 (2.9%)	0.416
**Charlson Score (without cancer and metastasis)**			0.413
Mean (SD)	0.61 (0.973)	0.53 (1.022)	
Median (range)	0 (0–7)	0 (0–4)	

Abbreviations: M, months, DM, diabetes mellitus, HTN, hypertension

### Overall survival

**Fig 2:**
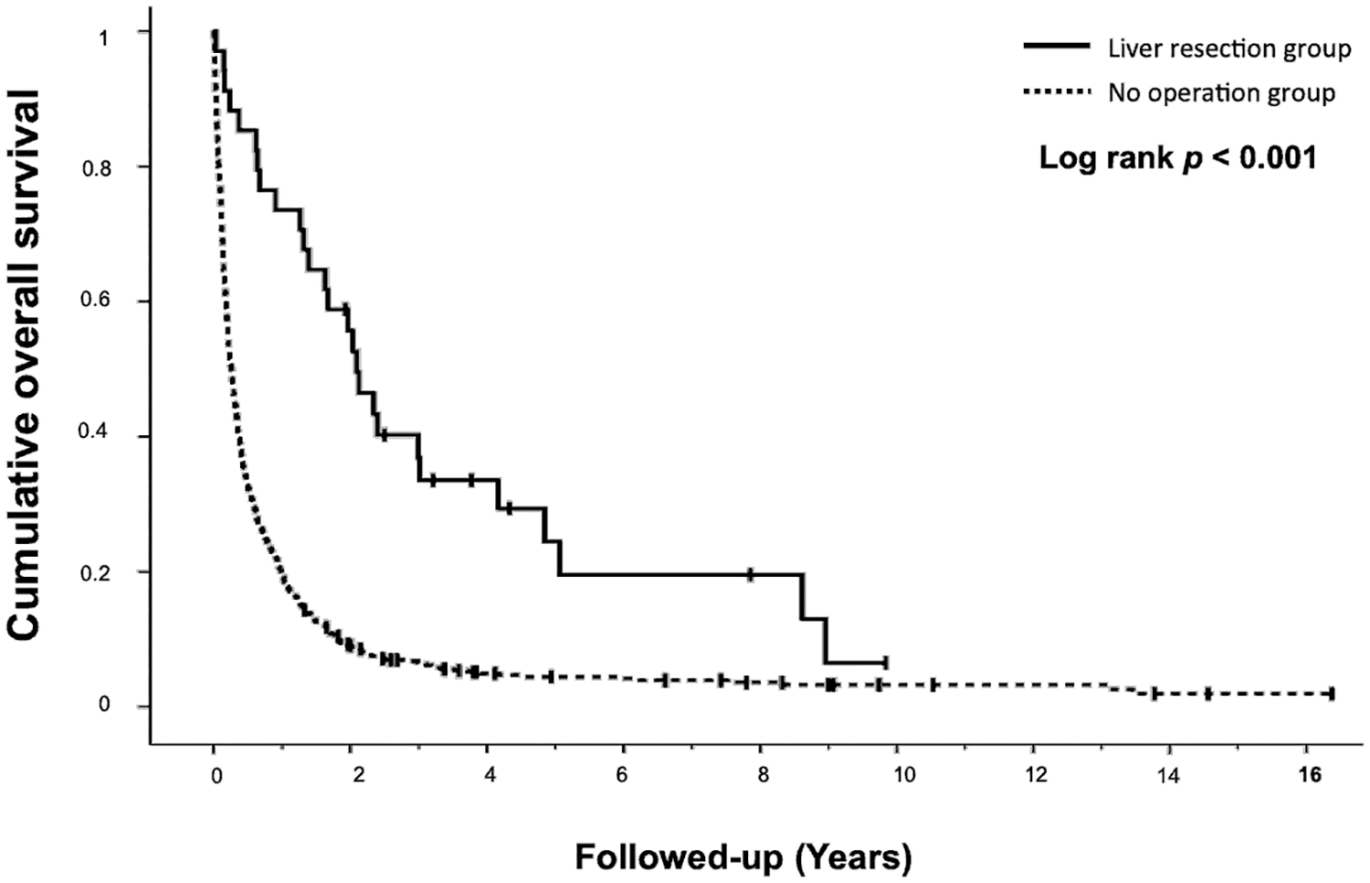
Overall survival following identification of liver metastasis in the surgical and non-surgical groups.

Fig 2 shows the result of Kaplan–Meier analysis with log-rank test for overall survival. Patients in the “liver resection” group had significantly better overall survival (longer median survival [26.16 vs 3.13 months] and higher 1-,2-,3-, and 5-year overall survival rates [73.5%, 55.7%, 36.9% and 24.5% vs 19.7%, 9.1%, 6.6%, and 4.4%; p<0.001]).

Entering age (p = 0.007) and liver resection or not (p < 0.001) into a COX regression model for multivariate analysis, showed that liver resection significantly increases the survival of patients with gastric cancer and metachronous liver metastasis (HR = 0.377, 95%CI: 0.255–0.556. p<0.001).

### Similarity of survival between patients treated before and after 2009

**Fig 3:**
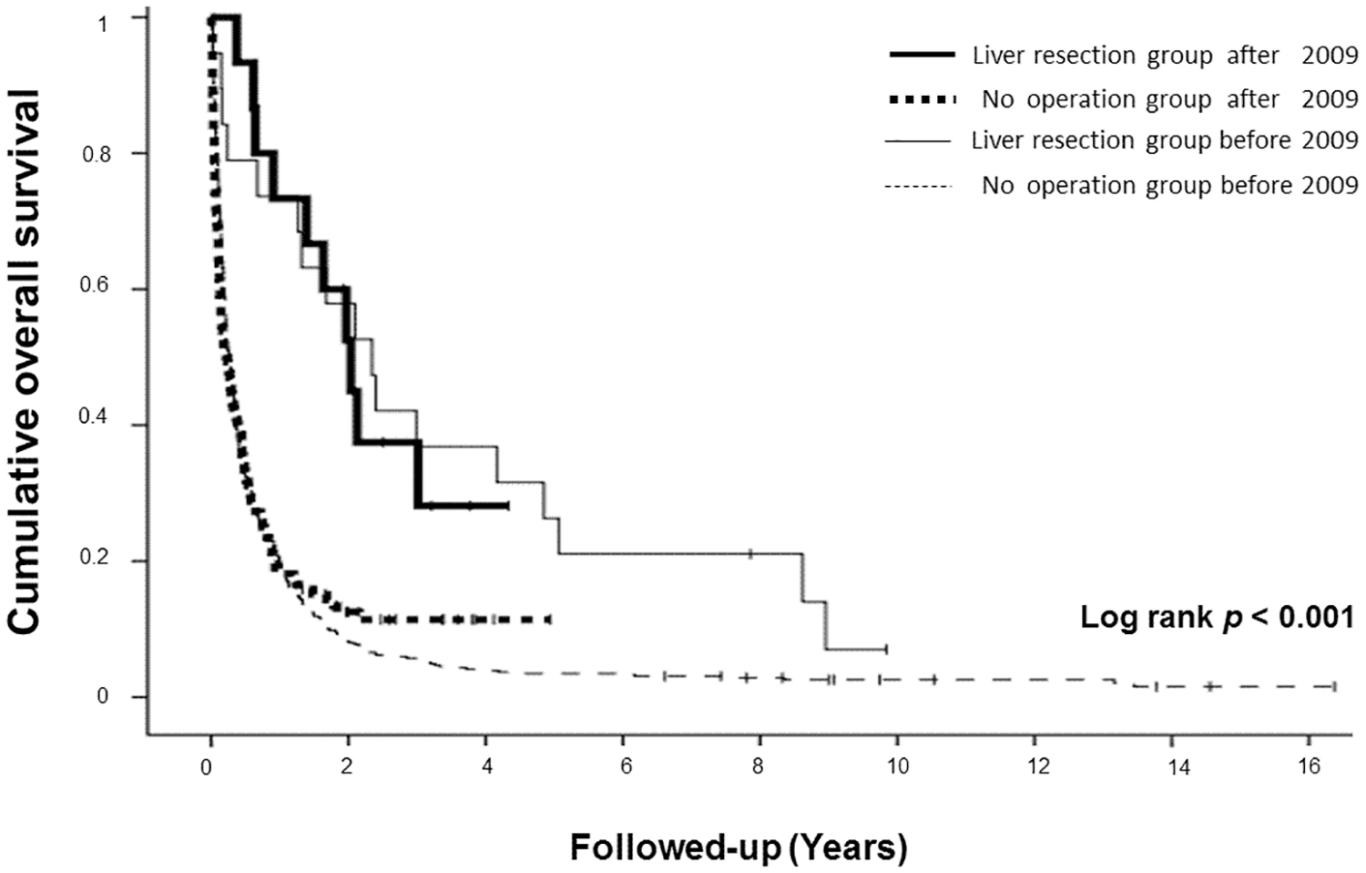
Overall survival following identification of liver metastasis in four different groups based on surgical status and surgical date (before vs after 2009).

We separated our patients into 4 groups, including two liver resection groups (one after and one before 2009) and two non-resection groups (one after and one before 2009) to evaluate survival difference before and after the introduction of the new regimen, Capecitabine and Oxaliplatin (XELOX). The patient numbers, overall survival, 1 year survival rate, and 3 year survival rate were listed in Table 2. Overall survival was significantly better in the liver resection group whether before 2009 (median survival 28.13 vs 3.23 months, p < 0.001) or after 2009 (median survival 24.43 vs 2.76 months, p = 0.001, Fig 3). However, median overall survival before 2009 and after 2009 was similar in the resection group (28.13 vs 24.43 months, respectively; p = 0.834) and non-resection group (3.23 vs 2.76 months, respectively; p = 0.698).

**Table 2:** Overall survival of resection group and non-resection group before and after 2009.

	Patients	Median overall survival (Months)	1 yr survival rate	3 yr survival rate
**(1) Resection group after 2009**	15	24.433	73.3%	37.5%
**(2) Non-resection group after 2009**	132	02.767	18.2%	11.4%
**(3) Resection group before 2009**	19	28.133	73.7%	36.8%
**(4) Non-resection group before 2009**	487	03.233	20.1%	05.5%

P value: (1) vs (2) = 0.001, (3) vs (4) <0.001, (1) vs (3) = 0.834, (2) vs (4) = 0.698

## Discussion

The present study found that resection of metachronous hepatic metastases from gastric cancer when compared to systemic treatment or palliation, with or without XELOX as salvage chemotherapy, prolonged overall survival. Our results suggest that liver resection should always be considered in cases of metachronous hepatic metastases from gastric cancer in the absence of secondary tumors or extra-hepatic metastases.

In our study, hepatic metastases were removed by local liver resection. There is no consensus on local treatment of metastatic lesions from gastric cancer, and a variety of therapies have been recommended by clinical practice guidelines, including radiofrequency ablation (RFA), transarterial chemoembolization (TACE), adjuvant chemotherapy, targeted molecular therapy, or palliative care[16–19]. A future prospective study to assess local treatment of gastric cancer with liver metastases will make it possible to plan specific randomized clinical trials to fully assess the effectiveness and benefit of local treatment for gastric cancer with liver metastases.

The main limitation of this study was our inability to restrict our analysis to a particular chemotherapy regimen. We were able to assess the impact of the XELOX regimen for metastatic gastric cancer on the basis of payment data in Taiwan’s National Health Insurance database since 2009. Our data showed that liver resection significantly improved the survival of patients with gastric cancer and metachronous liver metastases from gastric cancer both before and after 2009. So, even after aggressive chemotherapy for metastatic gastric cancer, liver resection for metachronous liver metastasis can still prolong survival.

Our study confirms that hepatic metastasis from gastric cancer is associated with a poor prognosis and long-term survival rarely occurs. However, after curative hepatectomy, our 1-,2-,3-, and 5-year overall survival rates were, respectively, 73.5%, 55.7%, 36.9%, and 24.5% with liver resection and 19.7%, 9.1%, 6.6%, and 4.4% without resection (p<0.001). After entering age (p = 0.007) and liver resection or non-resection (p < 0.001) as variables in a COX regression model, we conducted an analysis showing that liver resection provided significant survival benefit to patients with gastric cancer and metachronous liver metastasis (HR = 0.377, 95%CI: 0.255–0.556. p<0.001). The survival rate and median survival with liver resection were better than the median survival of 11.3 months and 1-year survival rate of 46% reported in REAL3 randomized controlled phase III trial, using epirubicin, oxaliplatin, and capecitabine (EOX)[20], or the median survival of 13.8 months reported in the ToGA randomized controlled phase III trial using trastuzumab [21].

Confounders including sex, utilization of neoadjuvant or adjuvant chemotherapy, and presence of peritoneal disease or non-liver metastases were not taken into consideration in our study and may have influenced some of the survival results presented. Survival rates are lower in the West than in the Far East and insufficient data are available to analyze more the survival differences between the Far East and Western studies[22].

### Limitations

There are other limitations in our study. First, selection bias is possible because the extent of tumor spread in the liver was not recorded in our database. Patients in the resection group may have presented with a more acceptable oncologic burden for surgical resection. Also, we used for analysis, the inpatient expenditures by admissions. Patients with liver metastasis who were treated in outpatient department was included. Although the absolute number of liver metastasis may be higher than the data presented here, we believe that the patients who needed admission should relatively had a more serious clinical condition. The data we presented still reflects clinical significance. Second, the timing, regimen, and dosage of chemotherapy were not recorded in the database and could not therefore be determined. Some patients might have received chemotherapy without being admitted. However, we believed that even in patients with metachronous liver metastasis who received aggressive chemotherapy for metastatic gastric cancer, liver resection still had a role.

Third, some details, including the initial actual stage, extent and pathological characteristics of the primary tumor, and the details of each operation were not recorded and therefore could not be obtained. Fourth, the data in this database are secondary and administrative in nature. There is a risk of miscoding since surgeons do not usually use ICD-9 coding but rather different coding and Health Insurance Surgical orders, which they obtain from the Taiwan NHI payment system. However, most ICD-9 codes during admission were assigned by professional coders based on the records during admission. Also, a code table comparing ICD-9 codes and NHI payment system codes has been generated by the National Health Insurance Administration Ministry of Health and Welfare. We consider that the rate of surgical procedure miscoding to be limited.

## Conclusion

Liver resection in patients presenting with metachronous hepatic metastases as the sole metastases after curative resection of gastric cancer is associated with a significant survival improvement and should be considered a treatment option for such patients. Specific randomized clinical trials to evaluate further the effectiveness of local treatment of gastric cancer with liver metastases should be planned.

## References

[1] Jemal A, Bray F, Center MM, Ferlay J, Ward E, Forman D. Global cancer statistics. CA Cancer J Clin. 2011;61(2):69–90. doi: 10.3322/caac.20107 . 21296855 10.3322/caac.20107

[2] Blot WJ, Devesa SS, Kneller RW, Fraumeni JF Jr. Rising incidence of adenocarcinoma of the esophagus and gastric cardia. JAMA. 1991;265(10):1287–9. . 1995976

[3] Kamangar F, Dores GM, Anderson WF. Patterns of cancer incidence, mortality, and prevalence across five continents: defining priorities to reduce cancer disparities in different geographic regions of the world. J Clin Oncol. 2006;24(14):2137–50. doi: 10.1200/JCO.2005.05.2308 . 16682732 10.1200/JCO.2005.05.2308

[4] Shin A, Kim J, Park S. Gastric cancer epidemiology in Korea. J Gastric Cancer. 2011;11(3):135–40. doi: 10.5230/jgc.2011.11.3.135 . 22076217 10.5230/jgc.2011.11.3.135 PMC3204471

[5] Saiura A, Umekita N, Inoue S, Maeshiro T, Miyamoto S, Matsui Y, et al Clinicopathological features and outcome of hepatic resection for liver metastasis from gastric cancer. Hepatogastroenterology. 2002;49(46):1062–5. . 12143202

[6] D'Angelica M, Gonen M, Brennan MF, Turnbull AD, Bains M, Karpeh MS. Patterns of initial recurrence in completely resected gastric adenocarcinoma. Ann Surg. 2004;240(5):808–16. doi: 10.1097/01.sla.0000143245.28656.15 . 15492562 10.1097/01.sla.0000143245.28656.15 PMC1356486

[7] Qiu JL, Deng MG, Li W, Zou RH, Li BK, Zheng Y, et al Hepatic resection for synchronous hepatic metastasis from gastric cancer. Eur J Surg Oncol. 2013;39(7):694–700. doi: 10.1016/j.ejso.2013.03.006 . 23579173 10.1016/j.ejso.2013.03.006

[8] Ambiru S, Miyazaki M, Ito H, Nakagawa K, Shimizu H, Yoshidome H, et al Benefits and limits of hepatic resection for gastric metastases. Am J Surg. 2001;181(3):279–83. . 11376587 10.1016/s0002-9610(01)00567-0

[9] Nio Y, Koike M, Omori H, Hashimoto K, Itakura M, Yano S, et al A randomized consent design trial of neoadjuvant chemotherapy with tegafur plus uracil (UFT) for gastric cancer—a single institute study. Anticancer Res. 2004;24(3b):1879–87. . 15274369

[10] Wagner AD, Unverzagt S, Grothe W, Kleber G, Grothey A, Haerting J, et al Chemotherapy for advanced gastric cancer. Cochrane Database Syst Rev. 2010;(3):CD004064 doi: 10.1002/14651858.CD004064.pub3 . 20238327 10.1002/14651858.CD004064.pub3

[11] Takemura N, Saiura A, Koga R, Arita J, Yoshioka R, Ono Y, et al Long-term outcomes after surgical resection for gastric cancer liver metastasis: an analysis of 64 macroscopically complete resections. Langenbecks Arch Surg. 2012;397(6):951–7. doi: 10.1007/s00423-012-0959-z . 22615045 10.1007/s00423-012-0959-z

[12] Wu CY, Chen YJ, Ho HJ, Hsu YC, Kuo KN, Wu MS, et al Association between nucleoside analogues and risk of hepatitis B virus-related hepatocellular carcinoma recurrence following liver resection. JAMA. 2012;308(18):1906–14. Epub 2012/11/20. . 23162861 10.1001/2012.jama.11975

[13] Markar SR, Mikhail S, Malietzis G, Athanasiou T, Mariette C, Sasako M, et al Influence of Surgical Resection of Hepatic Metastases From Gastric Adenocarcinoma on Long-term Survival: Systematic Review and Pooled Analysis. Ann Surg. 2016;263(6):1092–101. doi: 10.1097/SLA.0000000000001542 . 26797324 10.1097/SLA.0000000000001542

[14] Charlson M, Szatrowski TP, Peterson J, Gold J. Validation of a combined comorbidity index. J Clin Epidemiol. 1994;47(11):1245–51. Epub 1994/11/01. . 7722560 10.1016/0895-4356(94)90129-5

[15] Deyo RA, Cherkin DC, Ciol MA. Adapting a clinical comorbidity index for use with ICD-9-CM administrative databases. J Clin Epidemiol. 1992;45(6):613–9. Epub 1992/06/01. . 1607900 10.1016/0895-4356(92)90133-8

[16] Chen L, Song MQ, Lin HZ, Hao LH, Jiang XJ, Li ZY, et al Chemotherapy and resection for gastric cancer with synchronous liver metastases. World J Gastroenterol. 2013;19(13):2097–103. Epub 2013/04/20. doi: 10.3748/wjg.v19.i13.2097 . 23599631 10.3748/wjg.v19.i13.2097 PMC3623989

[17] Vogl TJ, Gruber-Rouh T, Eichler K, Nour-Eldin NE, Trojan J, Zangos S, et al Response to comment on "repetitive transarterial chemoembolization (TACE) of liver metastases from gastric cancer: local control and survival results": will there be clinical implications in the future? Eur J Radiol. 2013;82(9):1592–4. Epub 2013/05/07. doi: 10.1016/j.ejrad.2013.04.001 . 23642764 10.1016/j.ejrad.2013.04.001

[18] Ueda K, Iwahashi M, Nakamori M, Nakamura M, Naka T, Ishida K, et al Analysis of the prognostic factors and evaluation of surgical treatment for synchronous liver metastases from gastric cancer. Langenbecks Arch Surg. 2009;394(4):647–53. Epub 2008/03/18. doi: 10.1007/s00423-008-0311-9 . 18343941 10.1007/s00423-008-0311-9

[19] Jeurnink SM, Steyerberg EW, Hof G, van Eijck CH, Kuipers EJ, Siersema PD. Gastrojejunostomy versus stent placement in patients with malignant gastric outlet obstruction: a comparison in 95 patients. J Surg Oncol. 2007;96(5):389–96. Epub 2007/05/03. doi: 10.1002/jso.20828 . 17474082 10.1002/jso.20828

[20] Waddell T, Chau I, Cunningham D, Gonzalez D, Okines AF, Okines C, et al Epirubicin, oxaliplatin, and capecitabine with or without panitumumab for patients with previously untreated advanced oesophagogastric cancer (REAL3): a randomised, open-label phase 3 trial. Lancet Oncol. 2013;14(6):481–9. Epub 2013/04/19. doi: 10.1016/S1470-2045(13)70096-2 . 23594787 10.1016/S1470-2045(13)70096-2 PMC3669518

[21] Bang YJ, Van Cutsem E, Feyereislova A, Chung HC, Shen L, Sawaki A, et al Trastuzumab in combination with chemotherapy versus chemotherapy alone for treatment of HER2-positive advanced gastric or gastro-oesophageal junction cancer (ToGA): a phase 3, open-label, randomised controlled trial. Lancet. 2010;376(9742):687–97. Epub 2010/08/24. doi: 10.1016/S0140-6736(10)61121-X . 20728210 10.1016/S0140-6736(10)61121-X

[22] Markar SR, Karthikesalingam A, Jackson D, Hanna GB. Long-term survival after gastrectomy for cancer in randomized, controlled oncological trials: comparison between West and East. Ann Surg Oncol. 2013;20(7):2328–38. Epub 2013/01/24. doi: 10.1245/s10434-012-2862-9 . 23340695 10.1245/s10434-012-2862-9

